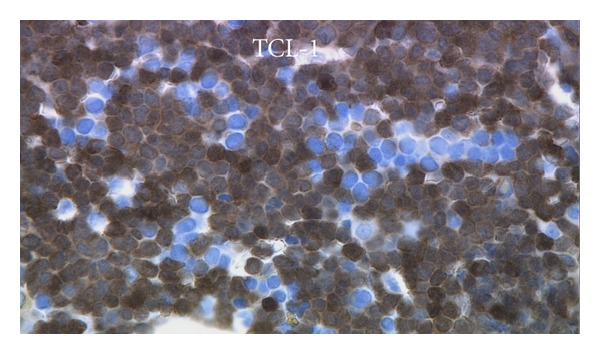# Good Clinical Response in a Rare Aggressive Hematopoietic Neoplasm: Plasmacytoid Dendritic Cell Leukemia with No Cutaneous Lesions Responding to 4 Donor Lymphocyte Infusions Following Transplant

**DOI:** 10.1155/2011/651906

**Published:** 2011-12-08

**Authors:** Amir Steinberg, Rina Kansal, Matthew Wong, Angela Lopez, Stephen Lim, Jean Lopategui, Michael Lill

**Affiliations:** ^1^Cedars-Sinai Medical Center, 8700 Beverly Boulevard, Los Angeles, CA 90048, USA; ^2^Division of Hematology-Oncology, Mount Sinai Medical Center, 1425 Madison Avenue, New York City, NY 10029, USA

## Abstract

Blastic Plasmacytoid Dendritic Cell Neoplasm (BPDCN) is a rare and aggressive malignancy that usually presents with diffuse cutaneous lesions. While a favorable response to therapy occurs in a majority of cases, a sustained long-term response is uncommon. Most patients subsequently relapse within a year. In the following report, we present the case of a 41-year-old woman who has not displayed many of the clinical features traditionally associated with BPDCN. The patient received sporadic chemotherapy treatment over the course of 2 years, before undergoing an allogeneic stem cell transplant. Although she ultimately relapsed following her transplant, her disease has repeatedly returned into remission after donor lymphocyte infusion (DLI). Currently, the patient is in remission following her fourth DLI. We believe that allogeneic transplantation should be considered as front-line therapy for the treatment of this rare malignancy.

## 1. Case Summary

A 41-year-old woman presented in December 2005 with fatigue, headache, tinnitus, nausea, and early satiety and was found to have abnormal blood counts. A bone marrow (BM) biopsy reportedly revealed undifferentiated acute leukemia (uAL). The patient was induced with a lymphoblastic AL regimen and was reported to be in remission at discharge in February 2006. She received no other chemotherapy until January 2007 when she presented again with leukopenia and recurrent leukemia. She first received 5 cycles of HyperCVAD. HyperCVAD consists of cyclophosphamide, vincristine, adriamycin, and dexamethasone alternating every 21–28 days with methotrexate and cytarabine along with intrathecal CNS prophylaxis. Each set of drugs is given four times for a total of eight cycles followed by maintenance [1]. She then developed thrombocytopenia and then received 6-mercaptopurine and methotrexate (Linker maintenance) for 10 months before her leukemia recurred in June 2008, with a BM biopsy again reported as uAL (WBC count = 0.8 × 10^9^/L, hemoglobin 7.6 g/dL, platelet count = 8 × 10^9^/L). She received induction chemotherapy and was referred to Cedars-Sinai Medical Center for transplantation consultation, after a BM biopsy in July 2008 showed no definitive evidence of residual leukemia. At Cedars-Sinai, she received 1 more cycle of HyperCVAD and underwent an HLA-matched sibling transplant in October 2008. In May 2009, a liver biopsy confirmed graft versus host disease (GVHD) and she received prednisone. She remained in remission until June 2010 when she again became cytopenic and BM biopsy showed relapsed uAL. Her immunosuppressive therapy was stopped, and she received Linker's cycle 1A and then high-dose Ara-C and mitoxantrone (HAM) to achieve remission. The Linker regimen consists of seven courses of therapy followed by maintenance [2]. Its chemotherapy differs from HyperCVAD in that prednisone is used instead of decadron, asparaginase is used, and 6-mercaptopurine is used in the courses of therapy as cyclophosphamide is not. She received a donor lymphocyte infusion (DLI) in August 2010. The cells for the DLI were obtained from the patient's sister on her initial stem cell harvesting and were stored at our hospital. There were three such additional bags stored. The first bag to be thawed and infused consisted of 1.12 × 10^8^ CD3 cells/kg, and the entire bag was given as a DLI. She was well for over 4 months with normal blood counts, apart from increased liver function tests (LFTs) suspected to be due to GVHD.

**Figure 1:**
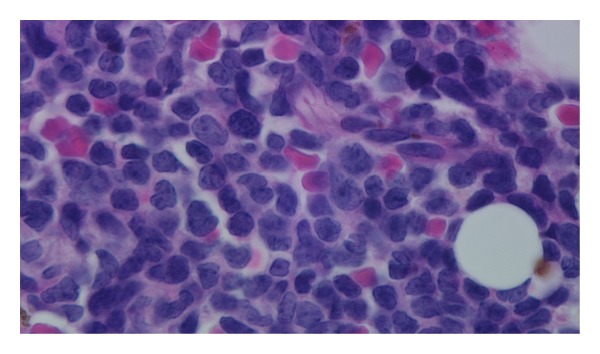


**Figure 2:**
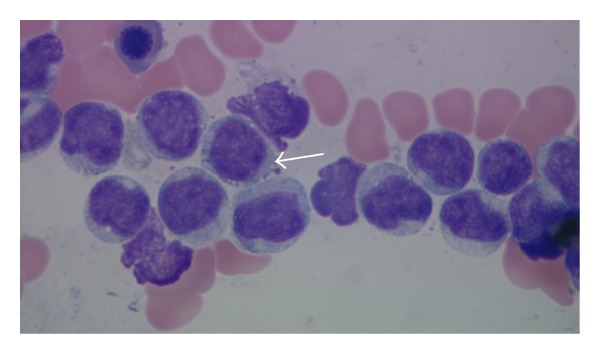


**Figure 3:**
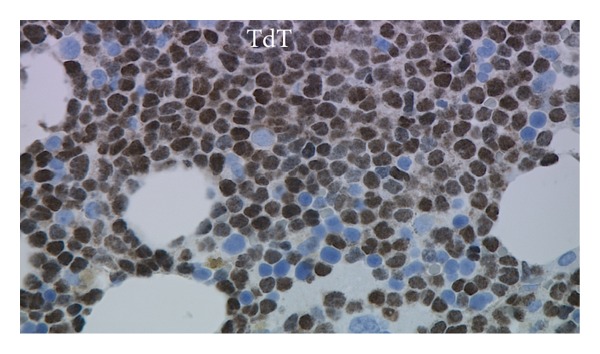


**Figure 4:**
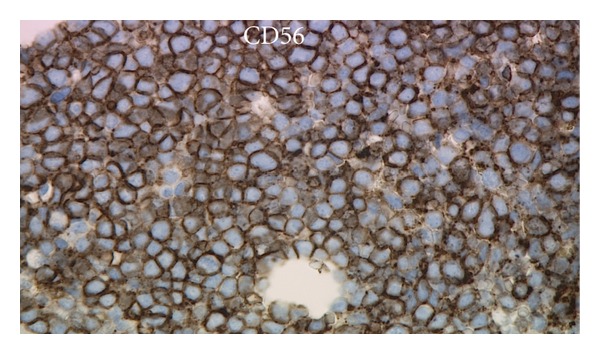


**Figure 5:**
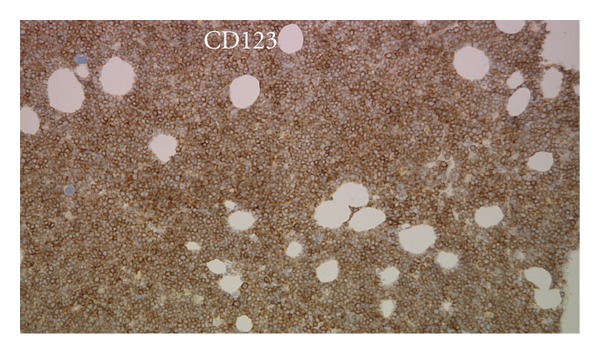


In late December 2010, she became thrombocytopenic, with normal LFTs, and showed circulating leukemic cells. The BM biopsy sections (Figure 1) showed tightly packed, variably sized, leukemic blasts with irregular nuclear contours or nuclear folds, finely dispersed chromatin, small, usually multiple nucleoli, and scant cytoplasm. The Wright-Giemsa-stained aspirate smears (Figure 2) showed blasts with irregular nuclear contours or nuclear folds, finely distributed chromatin, small nucleoli, and variably abundant, lightly basophilic, agranular cytoplasm with microvacuoles at the periphery of the cell (arrow in Figure 2). The leukemic cells were cytochemically negative for alpha naphthyl butyrate esterase. By flow cytometry, the leukemic cells were TdT^+^, bright CD56^+^, HLA-Dr^+^, dim CD45^+^, dim CD7^+^, dim CD38^+^, dim CD117^+^, CD3^−^, CD4^−^, CD5^−^, CD8^−^, CD10^−^, CD13^−^, CD14^−^, CD19^−^, CD20^−^, CD22^−^, CD23^−^, CD33^−^, CD34^−^, CD64^−^, *κ* ^−^, *λ* ^−^, cytoplasmic CD79a^−^, cytoplasmic CD3^−^, and cytoplasmic myeloperoxidase^−^. This immunophenotype was similar to the previous flow cytometric immunophenotype of the relapsed leukemia in June 2010. Immunohistochemical stains showed CD43^+^, TdT^+^ (Figure 3), CD56^+^ (Figure 4), CD123^+^ (Figure 5), and TCL-1^+^ (Figure 6) leukemic cells. T-cell receptor (TCR) T*γ*, IgH and Ig*κ* gene rearrangement (RA) showed several small clonal peaks likely representing the few admixed small T- and B-lymphocytes also detected by flow cytometry, similar to previous reports [3], while TCR T*β* gene RA was negative. Karyotypic analysis could not be performed. NPM1 exon 12 mutation, FLT3 ITD and FLT3 D835 mutation by PCR were negative. The above morphologic, immunophenotypic, and molecular findings led to a diagnosis of plasmacytoid dendritic cell (PDC) leukemia, as per the current WHO classification [4], wherein, before rendering a diagnosis of uAL, rare entities including PDC leukemia must be excluded. By cytomorphology, although no feature is considered specific for BPDCN, the blasts in this case are reminiscent of lymphoblasts with overlapping monocytic features and show peripherally situated microvacuoles, as typically described in this entity [5]. By immunophenotype, to diagnose by immunohistochemistry, the expression of at least 1 of 5 BPDCN-associated markers in a CD56^+^, CD4^±^, TdT^+^, lineage-negative neoplasm is considered minimal [5–7]. In this case, the leukemic cells were positive for 2 of 5 BPDCN-associated markers, CD123 and TCL-1.

The patient received cyclophosphamide, etoposide, and clofarabine (CEC) on 12/23/2010 for reinduction, and then received a DLI (containing 0.56 × 10^8^ CD3 cells/kg) on 12/29/10. Her blood counts normalized and her LFTs rose. She received an additional DLI on 2/21/2011 in order to maintain her graft-versus-leukemia effect. This was the third and final saved bag from the donor's original collection and contained 0.56 × 10^8^ CD3 cell/kg. Six weeks later, her blood counts dropped, LFTs normalized, and a BM biopsy confirmed relapsed PDC leukemia involving 50% of a 40% cellular marrow, with morphology and immunophenotype similar to the prior biopsy in December 2010. The patient received her 4th DLI on 5/6/11 (this one obtained from a recollection of the donor and containing 0.10 × 10^8^ CD3 cells/kg) after receiving CEC for reinduction.

## 2. Discussion

BPDCN is an entity recently included in the WHO classification [8]. This neoplasm was previously known as a CD4^+^CD56^+^ malignancy [7] and was shown to be derived from precursors of PDCs [7]. Patients with BPDCN usually present with asymptomatic skin nodules and plaques and invariably progress to acute leukemia [5]. Our case is unique since the patient did not have any skin lesions at any time during her illness and presented solely with leukemia. PDC leukemias are rare, comprising <1% of all leukemias [9], and examples without cutaneous involvement, similar to our case, are very rare [8]. In addition, our patient is a young female, while the typical BPDCN occur more commonly in males (M : F = 3.3 : 1) and in elderly patients, although patients may present at any age [4, 9–11].

 Therapeutically, a majority of BPDCN patients initially respond favorably; however, this response is short-lived and relapses occur within 9–11 months, on average [10–12], with median length of survival being 38 months for patients <40 years and only 10 months for those >40 years in age [12]. Long-lasting responses are rare, reported primarily in younger patients, with 2 of 21 patients reported in complete remission (CR) after 60 months following an allogeneic bone marrow transplant in first CR [10]. Older patients also respond favorably, as in a report of a 50-year-old male in remission 18 months after matched related stem cell transplant in first CR [13], and another of a 66-year-old with BPDCN that evolved into AML, in remission at 26 months without a transplant [14].

 The most compelling evidence for the efficacy of transplantation in BPDCN comes from a recent report of 6 elderly BPDCN patients (median age 67), 4 of whom received reduced-intensity allogeneic stem cell transplants, with 2 in remission at 57 and 16 months after transplant, respectively, [15, 16]. Our patient with purely leukemic BPDCN had remissions of reasonable duration in response to 4 DLIs and may be the first reported BPDCN patient to show a successful response to DLI, highlighting the efficacy of donor-versus-tumor effect in BPDCN. Due to the overall poor prognosis of BPDCN, allogeneic transplantation (myeloablative in young and reduced-intensity in older patients) should be considered as front-line therapy for BPDCN.

## Figures and Tables

**Figure 6:**